# A novel method to quantify the emission and conversion of VOCs in the smoking of electronic cigarettes

**DOI:** 10.1038/srep16383

**Published:** 2015-11-10

**Authors:** Yong-Hyun Kim, Ki-Hyun Kim

**Affiliations:** 1 Department of Civil and Environmental Engineering, Hanyang University, 222 Wangsimni-Ro, Seoul 133-791, Korea

## Abstract

An analytical technique was developed for the quantitation of volatile organic compounds (VOC) in three different forms of electronic cigarette (EC): solution, vapor, and aerosol. Through the application of the mass change tracking (MCT) approach, the consumed amount of the solution was measured to track the conversion of targets between the different phases. The concentration of aerosol plus vapor (A&V) decreased exponentially (559 to 129 g m^−3^) with increasing puff velocity (0.05 to 1 L min^−1^). A strong correlation existed between sampling volume and consumed solution mass (R^2^ = 0.9972 ± 0.0021 (n = 4)). In the EC solution, acetic acid was considerably high (25.8 μg mL^−1^), along with trace quantities of some VOCs (methyl ethyl ketone, toluene, propionic acid, and i-butyric acid: 0.24 ± 0.15 μg mL^−1^ (n = 4)). In the aerosol samples, many VOCs (n-butyraldehyde, n-butyl acetate, benzene, xylene, styrene, n-valeric acid, and n-hexanoic acid) were newly produced (138 ± 250 μg m^−3^). In general, the solution-to-aerosol (S/A) conversion was significant: e.g., 1,540% for i-butyric acid. The emission rates of all targets computed based on their mass in aerosol/ consumed solution (ng mL^−1^) were from 30.1 (p-xylene) to 398 (methyl ethyl ketone), while those of carboxyls were much higher from 166 (acetic acid) to 5,850 (i-butyric acid).

The use of electronic cigarettes (EC) is prevalent in Korea and other countries^1^,^2^. ECs are marketed as less harmful alternatives to tobacco smoking^3^. However, according to recent studies, many types of hazardous compounds (i.e., carcinogenic compounds) are emitted from EC smoke, some in significantly large quantities. For instance, some volatile organic compounds (VOC) like formaldehyde (FA) were generated in considerably large quantities upon puffing, as the EC solution was aerosolized via heating^4^,^5^. Therefore, information concerning the conversion of EC-related pollutants needs to be accurately assessed in order to better describe their potential damage to human health. To this end, studies that acquire or establish an analytical technique for the reliable quantitation of pollutants contained in EC solution, as well as those released directly to or generated newly by its smoking, should be prioritized.

In general, smoke samples of conventional tobacco cigarettes are analyzed using official methods such as those of the ISO and Canadian intense^6^. In the case of ECs, although some analysis methods have been proposed, they are poorly validated for general application to the quantitation of all different types of EC samples (i.e., liquid, vapor, and aerosol). Therefore, many researchers are investigating optimal methods for both the collection and analysis of EC samples. In general, EC refill solutions are analyzed using gas chromatography (GC) or liquid chromatography (LC) after the pretreatment of the EC solution (such as derivatization) or through dilution with an appropriate solvent(s)^7^,^8^,^9^,^10^. For EC vapor (or aerosol) samples, the analytical approaches that are comparable to the official method for conventional cigarettes have commonly been employed^11^. For example, EC aerosols can be absorbed on a solvent (or collected on a glass filter) and subjected to extraction in a certain medium. The extracted proportion is then analyzed using GC or LC systems^11^. However, there are large differences in the two cigarette types not only between the mechanisms of smoke generation (i.e., sensitized control of puff for EC vs. conventional cigarette), but also in the measurement methods used to determine their compositions. More specifically, in the case of ECs, the solvent effect must be considered, as the collected samples contain excessive quantities of propylene glycol (PG) and/or vegetable glycerin (VG). Unless treated properly, those solvents disrupt the detection of targets in the GC-based analysis. The direct application of conventional methods to EC smoke samples is unlikely to be feasible in many respects. Therefore, analytical methods should be developed to effectively accommodate the physicochemical characteristics of both the major and trace components of EC so that the generation mechanisms of those samples should be reflected properly in their quantitation.

The EC aerosols are generated from the EC refill solution in the electronically heated cartomizer (atomizer + cartridge). As the solvent of the EC liquid used primarily consists of PG and/or VG, it is important to consider two key factors: the solvent effect of these major components and the consumption rate of the EC solution. First, the presence of massive quantities of solvent (like PG and/or VG) in the EC solution disrupt the detection of target compounds, since they remain after thermal desorption^12^,^13^. In particular, the quantitative analysis of some target compounds with physicochemical properties similar to those of the solvent is hindered due to interference from chromatographic separation. Therefore, an analytical method that can effectively resolve this solvent effect (unlike tobacco cigarette) needs to be established. Second, the ECs can generate new compounds (e.g., formaldehyde, acetaldehyde, and acrolein) that did or did not exist in the original solution; they are generally produced via oxidation of the EC components (PG and VG) through heating^4^,^5^. The formation of those pollutants directly reflects the consumption of EC solution. Likewise, quantification of mass changes before and after the use of EC is more useful than only recording of the puffing conditions (e.g., puffing velocity, puffing duration, number of puffing, etc). This concept was thus defined as mass change tracking (MCT) approach. As a result, the mass balance between the amount of EC solution consumed by puffing and the amount of EC smoke caught up on the ST can be established to accurately assess mass transfer between different EC phases.

In the quantitative stage of this study, a total of 24 target VOCs in EC samples (either as aerosol phase or refill solution: refer to Table S1 in Supplementary Information. Note that acronyms are generally used for all target compounds throughout the text (e.g., benzene = B)) were treated identically not only during the sample collection (by a sorbent tube (ST)) but also during the instrumental detection (by the same GC-mass spectrometry (MS) system equipped with a thermal desorber (TD))^14^. As this combination of ST-TD-GC-MS approach allowed us considerably reduce sampling volume (e.g., EC liquid <3 μL and aerosol <10 mL), it helped us eliminate or effectively reduce the solvent effect between different sample types – between liquid and vapor phase samples. In addition, the solvent effect can be reduced further with the aid of purging^15^. The extracted ion chromatogram (EIC) and selected ion monitoring (SIM) modes of the MS system were applied to quantify the target VOCs at trace quantities^16^. The emission rates (ER) of the VOCs from the EC were calculated by simultaneously considering the consumption rate of the EC solution and the puff conditions of the EC smoke based on the optimal analytical approaches designed in this study.

## Results and Discussion

### Measurement of the sampling mass of the EC smoke (A&V) using the mass change tracking (MCT) method

In this study, the amount of EC solution consumed by puffing was measured based on the MCT approach, which allowed for the comparison of the consumed solution mass with the collected mass of the EC smoke on the ST. The difference between the two mass terms was almost negligible, at less than 3%. For this reason, the consumed amount of refill solution was used as a key index in the calculation of VOC emission rate from the EC device with varying puff conditions (Table 1).

**Figure 1:**
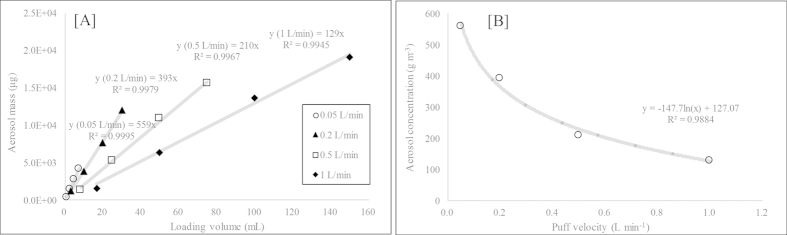
Emission trend of the VOCs in the form of EC aerosol in relation to sampling flow rate: Consumed amount of EC solution vs. puff conditions. (**A**) Aerosol sampling volume (mL) vs. consumed mass of EC solution (μg). (**B**) Aerosol concentration (g m^−3^) vs. aerosol sampling flow rate (L min^−1^).

The V_E_ concentration emitted from the EC was significantly low, at a value less than 1 g m^−3^, regardless of the puff conditions (total sample volume (0.01 to 2 L) and puff velocity (0.05 to 1 L min^−1^). In addition, the total amount (mg) of V_E_ collected on the sampler did not exhibit significant correlation with puff volume (L) (mean R^2^ ± SD = 0.2872 ± 0.2773 (n = 4)). As a result, when air was swept through the EC device filled with EC solution (without puffing; no operation of EC device), the amount of vaporized EC (V_E_) could be ignored in terms of mass. In contrast, the collection of A_E_ was accompanied by considerable changes in the mass quantities at all of the different puff conditions (puff velocity and volume). The emission mass of the A_E_ gradually increased with an increase in puff volume at a constant puff velocity (correlation (R^2^) between A_E_ mass vs. puff volume = mean 0.9972 ± 0.0021 (n = 4)). However, the concentrations of the A_E_ samples decreased logarithmically with an increase in puff velocity ((1) A_E_ concentration (puff velocity) = 559 g m^−3^ (0.05 L min^−1^) to 129 g m^−3^ (1 L min^−1^) and (2) R^2^ = 0.9888) (Fig. 1). The solvent of the EC solution consists of PG and VG. Because the boiling points of PG and VG were relatively high (188.2 and 290 °C, respectively), their conversion rates (solution to A&V) did not increase proportionally with the increases in the puff velocity.

### The results of the environmental EC samples (Exp. 1)

In this study, a total of 22 target VOCs in three types of EC samples (the EC solution (S_E_) and smoke (V_E_ and A_E_)) were analyzed using the ST-based method. In addition, acetic acid (ACA) was also quantified because of its abundance in all three types of EC samples. The quantitation of ACA was conducted using a linear regression analysis between carbon number and response factor (RF, ng^−1^) of seven of the target carboxyl compounds^17^,^18^,^19^. In the S_E_ analysis, five of the target VOCs (MEK, T, ACA, PPA, and IBA) were detected among all of the selected targets (Table S2). Except for ACA, the detected VOCs (n = 4) had low concentrations in the range of 0.059 (T) to 0.379 μg mL^−1^ (IBA) (Eq. (1)).


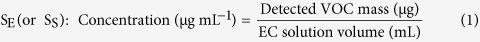


However, ACA was considerably high at 25.8 μg mL^−1^. In the case of V_E_, the VOCs (n = 10) with relatively low molecular weights (or high volatility) were also (or newly) detected (BA, MEK, BuAc, B, T, p,m,o-X, S, and ACA). The average concentration of the 10 VOCs detected from the V_E_ sample was 22.5 ± 51.8 μg m^−3^ (Eqn (2)).


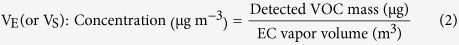


In the V_E_ sampling stage, the detection of light VOCs was expected due to their high volatility. In the A_E_ sample, all of the target VOCs detected from the S_E_ and V_E_ were present in greatly enhanced values relative to the other sample types (S_E_ and V_E_) studied concurrently.


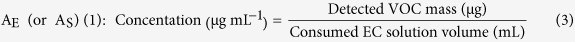



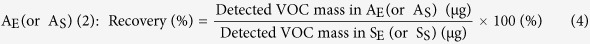


**Figure 2:**
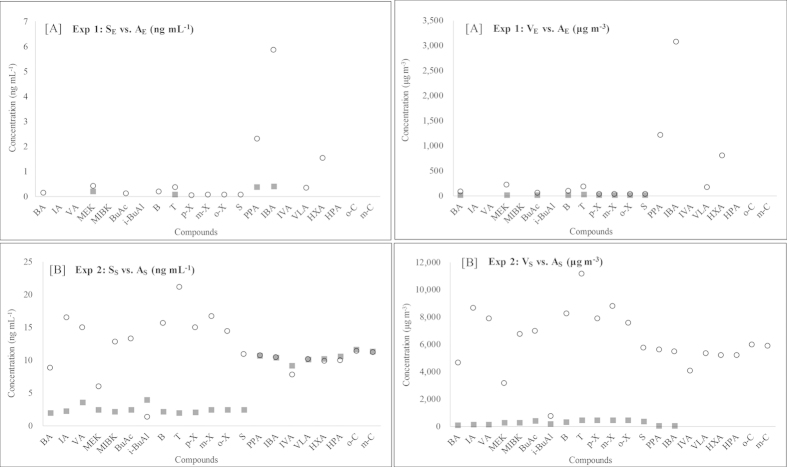
Comparison of the VOC concentrations between the EC solution (or vapor) and the aerosol samples (The former in filled rectangle and the latter in empty circle). (**A**) Exp 1: Analysis of the environmental EC samples. (**B**) Exp 2: Analysis of the spiked EC samples.

Therefore, the recovery of many of the VOCs in the S_E_ sample commonly exceeded 100% (232% for MEK: 0.40 μg mL^−1^ to 1,540% for IBA: 5.85 μg mL^−1^) (Eqs. (3) and (4)) (Fig. 2).

As a result, we confirmed that the VOC concentrations increased, especially in the case of carboxyl compounds; the solution-to-aerosol (S/A) conversion rates in these situations were 637% (PPA), 1,540% (IBA), and 646% (ACA)). In addition, two carboxyl compounds (VLA and HXA) that were not detected in the S_E_ were observed in the A_E_ with values of 0.31 μg mL^−1^ and 1.51 μg mL^−1^, respectively (Eq. (3)). The results of the V_E_ analysis were compared with those of the A_E_ based on the detected VOC mass (μg) per sample volume of V_E_ or A_E_ (L) (Eqs. (2) and (5)).


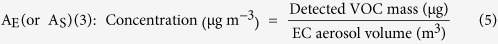



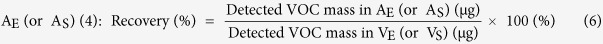


The recoveries of the six aromatic compounds in the A_E_ also increased considerably to 579% (o-X) −1,020% (B) (Eq. (6)): mean concentration ± SD = 57.5 ± 63.9 μg m^−3^ (n = 6) (Eq. (5)) (Fig. 2). The recoveries of BA, MEK, and BuAc in the A_E_ were 5,140%, 2,440%, and 1,300%, respectively, which were substantially larger than those of the V_E_ (Eq. (6)). The carboxyl compounds, PPA, IBA, VLA, and HXA, were not detected from the V_E_ but were detected in the A_E_ (mean concentration ± SD = 1,310 ± 1,250 μg m^−3^ (n = 4) (Eq. (5)). In the V_E_, ACA was measured at a moderately higher concentration of 169 μg m^−3^ (Eq. (5)) compared to the other simultaneously detected VOCs. The concentration of ACA increased significantly to 8.72E + 4 μg m^−3^ in the A_E_ (Eq. (5)), more than 500 times higher than that in the V_E_. Overall, the concentrations of most of the target VOCs increased considerably. It was striking to find that several of the carboxyl compounds were produced in large quantity through the conversion of S_E_ to A_E_. In addition, the VOCs with a relatively low boiling point (e.g., BP < 150 °C) were easily vaporized from the EC device filled with S_E_ when air was swept through the EC device without a puff.

### The results of the spiked EC samples (Exp. 2)

In order to provide a clear view of the conversion processes of the target VOCs across the three different EC sample types ((1) solution, (2) vapor, and (3) aerosol), analysis was also performed using the spiked refill solution (S_S_) containing all of the target VOCs. To this end, three different types of spiked samples were produced for a parallel analysis: (1) S_S_, (2) V_S_, and (3) A_S_. These samples were treated and analyzed in an identical manner to their environmental counterparts (S_E_, V_E_, and A_E_). The concentrations of the spiked EC samples were determined by treating the results of their corresponding environmental samples (S_E_, V_E_, and A_E_) as a blank. The changes in the VOC levels between the different sample phases can be examined thoroughly due to the availability of the data from these spiking samples.

The analysis of the S_S_ samples showed that the VOC recoveries were fairly variable depending on the functional groups present. For this comparison, the calibration results obtained by the L-WS were used as the basis for the recovery estimates. Accordingly, 13 of the target VOCs (except carboxyl (n = 7) and phenol compounds (n = 2)) had considerably lower recovery, with a mean of 38.7 ± 10.4% (n = 13) (Eq. (7)).


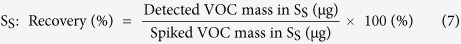


This low recovery of the 13 VOCs might have reflected the significant interfering effects of the EC solvents (PG or VG) or the unstable conditions of their mixtures in these solvents. In contrast, a nearly full recovery was achieved for the six carboxyl and two phenol compounds (mean recovery ± SD (n = 8) = 98.4 ± 4.17%) (Eq. (7)).

The vaporization efficiencies of the VOCs (from S_S_ to V_S_) were distinguished at least partially based on the physicochemical properties of each of the compounds. For example, the concentrations of the two carboxyl compounds (PPA and IBA) in the S_S_ were higher than those of the six aromatic compounds (mean concentration of S_S_: (1) two carboxyls = 10.5 ± 0.22 μg mL^−1^ and (2) six aromatics = 2.23 ± 0.22 μg mL^−1^) (Eq. (1)). However, in the V_S_, the aromatic compounds (n = 6) had noticeably higher concentrations, with a mean value of 386 ± 60.7 μg m^−3^ (n = 6), than those of the PPA and IBA with a mean value of 14.8 ± 0.17 μg m^−3^ (Eq. (2)). When the S_S_ was heated to produce the A_S_ sample, the emission patterns of the VOCs were clearly distinguished between the relatively light VOCs (BA, IA, VA, MEK, MIBK, BuAc, i-BuAl, B, T, p,m,o-X, and S (n = 13)) and the heavy VOCs (PPA, IBA, IVA, VLA, HXA, HPA, o-C, and m-C (n = 8)). In the case of the eight heavy VOCs (carboxyl and phenol compounds), their concentrations were similar between the A_S_ and S_S_, with a recovery of 96.6 ± 5.36% (n = 8) (calculated by Eq. (4)). The distribution of the heavy VOCs (n = 8) was comparable between the S_S_ and A_S_. In contrast, the concentrations of the light VOCs (n = 12), except for the i-BuAl, increased when the S_S_ was converted into A_S_ via puffing (246% (MEK) to 1,070% (T) (Eqn (4))). Therefore, most of those light VOCs can be very effectively converted from S_S_ to A_S_ in comparison to the heavy VOCs, such as carboxyl and phenol.

### Emission rates of the VOCs in the EC aerosol from the EC device

In this study, the emission rates of the VOCs from the EC were calculated in terms of ‘VOC mass released per puff duration’ in a direct connection to the consumption rate of the EC solution (S_E_). First, the consumption amount of the S_E_ was measured using the MCT approach at a puff velocity of 0.05 L min^−1^, while the VOCs aerosolized from the S_E_ were quantified using the ST/TD-GC-MS system. In addition, as the consumption rates of the S_E_ were measured at varying puff velocities of 0.2, 0.5, and 1 L min^−1^, the emission rates of the VOCs from the EC device with other puff flow rates (0.2, 0.5, and 1 L min^−1^) were also calculated using the results of the consumption trend of S_E_ at each puff velocity (Table 2).


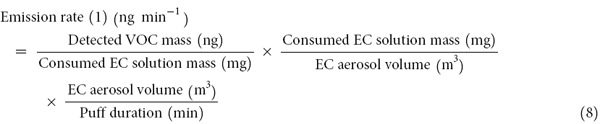


At the puff flow rate of 0.05 L min^−1^, six aromatic compounds and three VOCs (BA, MEK, and BuAc) had low mean emission rates of 3.64 ± 3.48 ng min^−1^ (n = 9) (Eq. (8)). In contrast, the emission rates of the five carboxyl compounds (PPA, IBA, VLA, HXA, and ACA) were relatively high (range of 7.89 (VLA) to 4,250 ng min^−1 (^ACA)) (Eq. (8)). The emission of the A_E_ sample from the EC device was evaluated depending on puff velocity (0.2, 0.5, and 1 L min^−1^) using the MCT approach. Based on these results, the emission rates of the VOCs from the EC device could be extrapolated at a puff velocity of 1 L min^−1^, which was comparable to the recommended puff velocity used for testing conventional cigarettes^6^.

The concentration levels of the VOCs in the EC aerosols measured by McAuley, *et al.*^11^ were quite comparable to those in our study. That previous study measured the VOCs in the EC aerosols emitted from EC solutions (n = 4) with tobacco flavor. To collect the EC aerosol samples, the EC solutions were aerosolized using an EC device equipped with a single cigarette smoking machine. Then, the EC aerosols were collected on the ST via a polyethylene bag. According to McAuley, *et al.*^11^, the emission concentrations of the VOCs were 219 ± 160 μg m^−3^ for BA, 180 ± 725 μg m^−3^ for VA, 28.2 μg m^−3^ for B, 244 ± 209 μg m^−3^ for T, and 107 ± 26.5 μg m^−3^ for p/m-X. It should be noted that our VOC results were 66.3 μg m^−3^ for BA, 87.5 μg m^−3^ for B, 176 μg m^−3^ for T, 15.7 μg m^−3^ for p-X, 26.5 μg m^−3^ for m-X, 18.8 μg m^−3^ for o-X, and 20.8 μg m^−3^ for S. In the case of a conventional cigarette, the emission rates of BTSX (mass per cigarette) generally ranged from 85 (p/m-X) to 1,100 (T) μg cigarette^−1^ 20,21,22,23,24,25. If it is assumed that it takes 20 puffs to consume a conventional cigarette, the average emission rate of the VOCs (mass per puff) was 16.3 ± 16.4 μg puff^−1^ (n = 6). If these results were compared with the data in this study, the EC counterparts of this study were significantly lower, with a mean of 0.43 ± 0.48 ng puff^−1^ (Eq. (9)).

## Conclusions

An electronic cigarette (EC) is a device designed to imitate tobacco cigarettes without combusting tobacco; therefore, the smoke (aerosol and vapor) is generated from the EC solution through electronic heating via a cartomizer. Therefore, it is important to assess the amount or exposure level of the hazardous compounds generated by EC puffing. In this study, VOCs in three types of EC samples (solution, vapor, and aerosol) were collected on the ST and analyzed using the same TD-GC-MS system to allow a parallel comparison. In addition, the consumption rates of the EC solution (S_E_) were evaluated with respect to puff conditions, so that the emission rates (VOC mass per puff duration) of the VOCs were compared between diverse criteria (per puff or per solution consumed).

In the analysis of the S_E_, MEK, T, PPA, and IBA were detected in low concentrations (mean 0.24 μg mL^−1^), while ACA was observed at a relatively higher concentration of 25.8 μg mL^−1^. The seven VOCs with relatively high volatility (BA, BuAc, B, p-X, m-X, o-X, and S) were also detected from the EC vapor sample (V_E_) without a puff. In the EC aerosol sample (A_E_), all of the VOCs detected from the S_E_ and V_E_ were observed at much larger quantities. In addition, two carboxyl compounds (VLA and HXA), which were not detected from the S_E_ and V_E_, were also produced. The concentrations of all of the VOCs detected from the A_E_ sample were considerably increased compared to those of the S_E_ (2.32 (MEK), by 15.4 times (IBA)), or the V_E_ (5.79 (o-X), by 515 times (ACA)). If the A_E_ sample was produced and inhaled at 1 L min^−1^, then approximately 20 ng of benzene (B) for one minute puff duration can be inhaled. In addition, the carboxyl compounds, such as the PPA, IBA, VLA and HXA, were also inhaled with a relatively high concentration of approximately 300 ng for one minute puff duration.

In this study, we developed an analytical method for the accurate quantitation of all types of EC samples (solution, vapor, and aerosol) based on ST sampling and TD-GC-MS analysis. Using the quantitative results of the EC samples, the emission rates of VOCs from an EC device were calculated based on the MCT approach in direct association with the puff conditions. The versatile applicability of this proposed approach was determined to be sufficiently efficient to accurately assess the emission properties of VOCs from all different types of EC samples. In addition, this method was sufficiently effective enough to estimate the emission factor through the direct quantitation of the diverse target analytes (transferred or converted) among the different EC phases with the aid of MCT approach.

## Materials and Methods

### Experimental outline

In this study, an experimental method for the accurate analysis of three types of EC samples (solution, vapor, and aerosol) was developed using the ST/TD-GC-MS system. The changes in concentration of target analytes in the EC samples were measured using the MCT approach with varying puff conditions of puff velocity, puff duration, and puff volume. As the consumption rate of the EC smoke was measured simultaneously at each of the smoke sampling stages, the VOC emission rates (or concentration) were presented in a number of ways using the detected mass of the target (in ng (or μg)) against per puff (mass per puff (ng puff^−1^), per puff duration (ng min^−1^), per air (or gas) collected (ng m^−3^ (air)), or per solution consumed (ng mL^−1^ (solution)). Therefore, our approach allows for the parallel comparison of all of the different concepts and in associated unites.

The calibration and quality assurance (QA) data for the target VOCs were obtained by analyzing their liquid working standard with the ST/TD-GC-MS system (Table S3). The three types of EC samples (solution, vapor, and aerosol) were then quantified using these calibration results. For this experiment, we used the EC device (Korea) and an EC solution without nicotine (Korea) (Table S4). Our experiments were then conducted in two different stages (Table 3). In Exp. 1, three different forms of the environmental EC samples (subscript of E) were collected and analyzed: (1) (refill) solution (S_E_), (2) vapor (V_E_) collected during the interpuff period (without puffing), and (3) aerosol (A_E_) plus V_E_ collected simultaneously due to puffing (It should be noted that aerosol alone cannot be sampled as both aerosol and vapor are released during puffing. The aerosol data were hence obtained by subtracting the vapor fraction from the A&V concentration). In Exp. 2, a spiked EC solution was prepared by adding the liquid working standard of the target VOCs into the S_E_. As all of the types of EC samples generally contained little or none of most of the target VOCs, their partitioning patterns were analyzed in detail using these spiked samples (with a subscript of S). The spiked EC solution (S_S_) and spiked A&V (A_S_ and V_S_) samples were then analyzed using the system introduced above. Based on the analysis of the spiked samples, we sought explanations for the generation and/or conversion patterns of the various targets in EC solution and in EC smoke. A detailed description of the liquid working standard preparation for quantitation of EC samples (Table S5), instrumental system (Table S6), and the sampling approaches of three types of EC samples (Figure S1) is provide in Supplementary Information (SI). In addition, the representative chromatograms of carboxyl compounds with different MS detection mods (total ion chromatogram (TIC) and selected the spectrum (SIM)) are shown in Figure S2.

## Additional Information

**How to cite this article**: Kim, Y.-H. and Kim, K.-H. A novel method to quantify the emission and conversion of VOCs in the smoking of electronic cigarettes. *Sci. Rep.* **5**, 16383; doi: 10.1038/srep16383 (2015).

## Supplementary Material

Supplementary Information

## Figures and Tables

**Table 1:** The mass change in EC smoke (A_E_ and V_E_, A&V) from the environmental samples according to puff velocity.

[A] Mass of the V_E_ and A_E_ samples								
Order	Puff velocity (L min^−1^)	(mL sec^−^1)	V_E_			A_E_		
			Loading time (sec)	Loading volume (L)	Mass (mg)	Loading time (sec)	Loading volume (mL)	Mass (mg)
1	0.05	0.83	12	0.01	0	1	0.8	0.4
2			24	0.02	0.1	3	2.5	1.4
3			60	0.05	0	6	5	2.8
4			120	0.1	0.1	9	7.5	4.2
5	0.2	3.33	12	0.04	0.2	1	3.3	1.2
6			24	0.08	0	3	10	3.8
7			60	0.2	0	6	20	7.6
8			120	0.4	0.3	9	30	12
9	0.5	8.33	12	0.1	0	1	8.3	1.3
10			24	0.2	0	3	25	5.2
11			60	0.5	0	6	50	10.9
12			120	1	0.2	9	75	15.6
13	1	16.7	12	0.2	0	1	16.7	1.5
14			24	0.4	0.1	3	50	6.3
15			60	1	0	6	100	13.6
16			120	2	0.1	9	150	19.1
**[B] Emission trends of the V**_**E**_ **and A**_**E**_ **samples in terms of consumed amount of EC solution**								
**Order**	**Puff velocity (L min**^**−1**^)	**V**_**E**_				**A**_**E**_		
		**Concentration (g m**^**−3**^)		**R**^**2**^		**Concentration (g m**^**−3**^)	**R**^**2**^	
1	0.05	0.923		0.1077		559	0.9995	
2	0.2	0.615		0.2410		393	0.9979	
3	0.5	0.154		0.6923		210	0.9967	
4	1	0.046		0.1077		129	0.9945	

**Table 2:** The emission rates of the VOCs in the A_E_ samples.

Order	Puff velocity (L min^−1^):	Emission rates^a^								
		VOC (ng)/puff duration (min)					VOC (ng)/puff number			
		0.05	0.2	0.5	1		0.05	0.2	0.5	1
1	BA	3.23	9.08	12.2	15.0		0.11	0.30	0.41	0.50
2	MEK	10.2	28.5	38.2	47.0		0.34	0.95	1.27	1.57
3	BuAc	2.50	7.03	9.41	11.6		0.08	0.23	0.31	0.39
4	B	4.26	12.0	16.0	19.7		0.14	0.40	0.53	0.66
5	T	8.60	24.1	32.3	39.8		0.29	0.80	1.08	1.33
6	p-X	0.77	2.15	2.88	3.55		0.03	0.07	0.10	0.12
7	m-X	1.30	3.65	4.89	6.01		0.04	0.12	0.16	0.20
8	o-X	0.92	2.58	3.45	4.25		0.03	0.09	0.12	0.14
9	S	1.01	2.84	3.80	4.68		0.03	0.09	0.13	0.16
10	PPA	58.4	164	220	270		1.95	5.47	7.32	9.01
11	IBA	149	419	561	691		4.98	14.0	18.7	23.0
12	VLA	7.89	22.2	29.7	36.5		0.26	0.74	0.99	1.22
13	HXA	38.4	108	145	178		1.28	3.60	4.82	5.93
14	ACA	4,250	1.19E+4	1.60E+4	1.97E+4		142	397	532	655

^a^

**Table 3:** Basic information on the puff conditions in order to collect the EC samples depending on sampling approach^a^.

Order	Sample code		Liquid sampling conditions^b^		Smoke sampling conditions^c^		Aerosol generation conditions^d^					Consumed amount of EC solution for the analysis^e^	
		Sample phase	Purging time (min)	Purge volume (L)	Sampling time (min)	Sample volume (L)	EC puffing	Puffing duration (sec)	Puff number	Sampling time (min)	Sample volume (mL)	Volume (μL)	Mass (mg)
**[A] Exp. 1: Analysis of the environmental EC samples**													
1	S_E_	Liquid	5	0.25	—	—	—	—	—	—	—	1	1.096
2	V_E_(1)	Vapor	—	—	0.2	0.01	—	—	—	—	—	—	—
3	V_E_(2)	"	—	—	0.4	0.02	—	—	—	—	—	—	—
4	V_E_(3)	"	—	—	1	0.05	—	—	—	—	—	—	—
5	V_E_(4)	"	—	—	2	0.1	—	—	—	—	—	—	—
6	A_E_(1)	Aerosol	—	—	0.4	0.02	O	1	1	0.02	0.83	0.36	0.4
7	A_E_(2)	"	—	—	0.4	0.02	O	3	1	0.05	2.5	1.28	1.4
8	A_E_(3)	"	—	—	0.4	0.02	O	3	2	0.1	5	2.55	2.8
**[B] Exp. 2: Analysis of the EC sample spiked with liquid VOC working standard**^f^													
9	S_S_	Liquid	5	0.25	—	—	—	—	—	—	—	0.5	0.548
10	V_S_(1)	Vapor	—	—	0.2	0.01	—	—	—	—	—	—	—
11	V_S_(2)	"	—	—	0.4	0.02	—	—	—	—	—	—	—
12	V_S_(3)	"	—	—	1	0.05	—	—	—	—	—	—	—
13	V_S_(4)	"	—	—	2	0.1	—	—	—	—	—	—	—
14	A_S_(1)	Aerosol	—	—	0.4	0.02	O	1	1	0.02	0.833	0.36	0.4
15	A_S_(2)	"	—	—	0.4	0.02	O	3	1	0.05	2.5	1.28	1.4
16	A_S_(3)	"	—	—	0.4	0.02	O	3	2	0.1	5	2.55	2.8

^a^The sampling (or purge) flow rate for all of the experiments was fixed at 0.05 L min^−1^.

^b^Liq: The EC liquid sample was directly injected into the sorbent tube, while filtered ambient air (by Carbopack X) flowed to the sorbent tube.

^c^Smoke: Smoke was used to represent both the vapor and aerosol fractions of EC samples. In the case of the vapor samples, the air was swept through the EC device filled with the liquid sample for vaporization without puffing. The swept vapor samples were collected on the sorbent tube.

^d^Aer: The EC liquid sample filling the EC device was ‘aerosolized’ during the EC operation. The aerosol samples were collected on the sorbent tube.

^e^In the case of the aerosol samples, the amount was the consumed mass of the EC solution during the aerosol sampling.

^f^0.2 μL of both the PS-1 and PS-2 was spiked into the EC liquid sample in order to obtain the 2 mL spiking solution.
