# Supplementary material for: A novel method to quantify the emission and conversion of VOCs in the smoking of electronic cigarettes
Source: Sci Rep. 2015 Nov 10;5:16383. doi: 10.1038/srep16383 (PMC4639842; doi:10.1038/srep16383)
Supplement: Supplementary Information [file srep16383-s1.pdf]

Supporting Information

A novel method to quantify the emission and conversion of VOCs in the smoking of electronic cigarettes

Yong-Hyun Kim, Ki-Hyun Kim*
Department of Civil and Environmental Engineering, Hanyang University,
222 Wangsimni-Ro, Seoul 133-791, Korea

Keywords: electronic cigarette, emission rate, volatile organic compound

* Corresponding author: kkim61@hanyang.ac.kr or kkim61@nate.com, Phone: 82-2-2220-2325. Fax -1945

Materials and methods

Preparation of the liquid working standards and electronic cigarette spiking solution. A total of 22 volatile organic compounds (VOCs) were selected as the primary target analytes in this study: (1) three aldehydes: n-butyraldehyde (BA), i-valeraldehyde (IA), and n-valeraldehyde (VA); (2) two ketones: methyl ethyl ketone (MEK) and methyl isobutyl ketone (MIBK); (3) ester: n-butyl acetate (BuAc); (4) alcohol: i-butyl alcohol (i-BuAl); (5) six aromatics: benzene (B), toluene (T), p-xylene (p-X), m-xylene (m-X), o-xylene (o-X), and styrene (S); (6) seven carboxyls: propionic acid (PPA), i-butyric acid (IBA), n-butyric acid (BTA), i-valeric acid (IVA), n-valeric acid (VLA), n-hexanoic acid (HXA), and n-heptanoic acid (HPA); and (7) phenol: o-cresol (o-C) and m-cresol (m-C) (Table S2). For the reader's reference, all of these target VOCs except benzene have been shown to have considerably low odor thresholds[1-4].

The reagent grade chemicals (RGC) containing the 22 target VOCs were purchased at a purity ≥ 97.0% (Sigma-Aldrich, USA). The primary standards (PS) were prepared separately in PS-1 and PS-2 by adding some amount of each RGC: (1) PS-1 (Group 1, n=13): aldehydes (n=3), ketone (n=2), ester (n=1), alcohol (n=1), and aromatics (n=6); and (2) PS-2 (Group 2, n=9): carboxyls (n=7) and phenols (n=2). The VOCs of PS-1 were moderately or highly volatile, while the VOCs of PS-2 consisted of semi-volatile compounds. The mean concentrations of PS-1 and PS-2 were 64.2 ± 3.27 μg μL^{-1} and 106 ± 5.22 μg μL^{-1}, respectively. The first liquid working standard (1st L-WS) was made by mixing 4 μL of each of the PS (PS-1 and -2) with 3,992 μL of methanol (≥ 99.8%, Burdick & Jackson, USA) in order to obtain a 4 mL volume (mean ± SD concentration: 81.4 ± 21.7 ng μL^{-1}). The final L-WS for the four-point calibrations was prepared

through the dilution of the first L-WS with methanol in order to generate four different concentration levels (mean ± SD concentration: 4.07 ± 1.09 (1st) to 81.4 ± 21.7 ng μL^{-1} (4th point) (Table S4). The spiked EC solution (S_S) for Exp. 2 was prepared by spiking 0.2 μL of both PS-1 and PS-2 into 1,999.6 μL of the S_E in order to obtain a 2 mL volume. The mean concentration (± SD) of the S_S was 8.14 ± 2.17 ng μL^{-1}.

Instrumental system. All of the analyses in this study were carried out using a GC (Shimadzu GC-2010, Japan) equipped with an MS (Shimadzu GCMS-QP2010 Ultra, Japan) and a TD (UNITY II, Markes International, Ltd., UK). The TD focusing trap was prepared by packing Carbopack C and B in a 1:1 volume ratio (ID of the trap holder: 2 mm and total sorbent bed length: 50 mm) (Table S5).

The STs were prepared identically by packing Carbopack-C (70 mg), -B (50 mg), and-X (50 mg) (Supelco, USA) into an empty quartz holder[5]. A multi-bed ST has been demonstrated to yield a high recovery of VOCs[6]. The VOCs loaded onto the ST were thermally desorbed, transferred to the GC, and separated on a CP-wax column (diameter: 0.25 mm, length: 60 m, and thickness: 0.25 μm) for MS detection.

To minimize the interference effect by EC solvent (PG and/or VG), the SIM and EIC modes of the MS system were used to detect the target VOCs[7]. The VOCs (Group 2 (n=9): carboxyl and phenol) with a retention time close to that of the PG or VG were more sensitively affected by the solvent effects. For this reason, the chromatograms of the nine VOCs in Group 2 were obtained in SIM mode. In the case of Group 1 (n=13: aldehyde, ketone, ester, alcohol, and aromatic), the solvent effect was insignificant due to the large differences in retention times compared to that of the EC solvent. However, in order to eliminate such an

effect, EIC mode was used for quantitation of these VOCs. These Group 1 VOCs were initially examined in total ion chromatogram (TIC) mode over a mass range of 35 to 500 m/z. Then, EIC mode was subsequently applied using the information for the identified ions based on the spectrum of each of the VOCs (Table S3).

Calibration of the liquid working standard and the analysis of the two types of EC solution: Environmental (S_E) and spiked (S_S) samples. The inlet and outlet of the ST were connected to a filter tube packed with Carbopack X (100 mg) and the vacuum pump interfaced with the mass flow controller (Shibata ΣMP-30, Japan), respectively. For the analysis of the L-WS, S_E, and S_S, each of these substances was injected onto the ST by piercing through a temporary injection port made of silicone tubing used to hold the Teflon tube that connected the inlet of the ST and the filter tube. In this experiment, filtrated air was constantly supplied through the Carbopack X tube (purge flow rate of 50 mL min^{-1} for 5 min)[6]. The ST-loaded samples were then analyzed using the TD-GC-MS system (Figure S1 and Table 3).

Analysis of electronic-cigarette ‘vapor’ and ‘aerosol plus vapor (A/V)’. The inlet of the ST was initially connected to the tips of the EC device filled with 2 mL of EC solution (S_E or S_S) via Teflon tubing. The outlet of the ST was then connected to the vacuum pump (Shibata ΣMP-30, Japan) using silicone tubing. For collection of vapor samples (V_E or V_S), external air was pulled through the EC device (filled with EC solution) on the ST without puffing at a fixed flow rate of 0.05 L min^{-1} for four different loading volumes

of 0.01, 0.02, 0.05, and 0.1 L. In the aerosol (plus vapor: A/V) sampling, the EC solution filling the EC device was aerosolized under three types of puffing conditions (puff duration and puff number: (1) 1 sec and 1 time, (2) 3 sec and 1 time, and (3) 3 sec and 2 times), while the air was swept through the EC device using the vacuum pump (at a fixed flow rate of 0.05 L min^{-1} for 0.4 min). In the quantitation of the A/V sample, the concentration of the solely aerosol fraction (A_E or A_S) was computed by treating the vapor fraction (V_E or V_S) as a blank (Figure S1 and Table 3).

Mass change tracking (MCT) of the EC samples. To apply the MCT approach, the emission amount of EC smoke (A/V) was evaluated by simultaneously considering puff conditions (puff velocity and puff duration) and solution consumption rate. This information was then used to calculate the VOC emission rate, such as the mass of the pollutant released per puff or per consumed amount of solution (in mass or volume). To this end, the consumed mass (from the EC solution) and the collected mass (on the ST) were simultaneously measured during EC sampling.

To measure the consumption rate of EC solution, the inlet and outlet of the ST (for the scrubber) were connected to a tip of the EC device filled with 2 mL EC solution (S_E) and the vacuum pump. Teflon tubing was used to connect the ST and the EC device at one end and the vacuum pump at the other. In the case of the vapor sample, four different flow rates of 0.05, 0.2, 0.5, and 1 L min^{-1} were applied at four different sampling times of 0.2, 0.4, 1, and 2 min. Weight from both the ST loaded with vapor sample and the EC device was measured both before and after vapor sampling using a scale (HM-202, A&D, Japan). Likewise,

the sampling flow rates (puff velocity) for the aerosol sample were set at 0.05, 0.2, 0.5, and 1 L min^{-1} with sampling times (puff duration) of 1, 3, 6, and 9 sec (puff duration and puff number (total sampling time): (1) 1 sec and 1 time (1 sec), (2) 3 sec and 1 time (3 sec), (3) 3 sec and 2 times (6 sec), and (4) 3 sec and 3 times (9 sec)). The weights of the ST and EC device were also measured at each interval. As a result, the aerosol emission amount due to the EC operation with different puff conditions (puff velocity, duration, and numbers) were evaluated in reference to the consumed amount of EC solution using the MCT approach.

Calibration and quality assurance (QA) of the ST/TD-GC-MS analysis of the EC samples. The basic calibration and QA data for the 22 target VOCs were obtained through the analysis of the L-WS using the ST/TC-GC-MS system (Table S1). All of the target VOCs had fairly good linearity (mean R^2 ± SD = 0.9957 ± 0.0017 (n=22)) according to the ST-based analysis of the liquid VOC standards. In addition, the reproducibility of the ST method was assessed in terms of relative standard error (RSE, %) using triplicate analyses of the second calibration point (19.4 ± 5.17 ng μL^{-1} (n=22)) of the final L-WS. The RSE values of all of the target VOCs (n=22) were low, with a mean (± SD) of 1.18 ± 0.68%. The lowest detectable amount of each target VOC (n=22) was calculated as the method detection limit (MDL) according to the relevant U.S. EPA guidelines [7]. Seven repeated analyses were performed using the standard of the mean (± SD) of 0.41 ± 0.11 ng μL^{-1}, which was obtained by diluting the final L-WS of the first calibration point (Table S1). The resulting SD values were then multiplied by 3.14 (Student's t-value at the 99.9% confidence interval) in order to yield the MDL in mass quantity (ng), which ranged from 0.014 ng (styrene, S) to 0.197 ng (PPA)

(mean MDL ± SD (n=22) = 0.055 ± 0.039 ng). If the puffing sample volume of the EC smoke (aerosol or vapor) was assumed to be 0.01 L, the MDL value equated to an approximately 1 ppb level of the VOCs.

References

1. Karpe, P., Kirchner, S. & Rouxel, P. Thermal desorption-gas chromatography-mass spectrometry-flame ionization detection-sniffer multi-coupling: A device for the determination of odorous volatile organic compounds in air. *J. Chromatogr. A* **708**, 105-114 (1995).
2. Dincer, F., Odabasi, M. & Muezzinoglu, A. Chemical characterization of odorous gases at a landfill site by gas chromatography–mass spectrometry. *J. Chromatogr. A* **1122**, 222-229 (2006).
3. Saral, A., Demir, S. & Yıldız, Ş. Assessment of odorous VOCs released from a main MSW landfill site in Istanbul-Turkey via a modelling approach. *J. Hazard. Mater.* **168**, 338-345 (2009).
4. Kim, Y.-H. & Kim, K.-H. A test on the reliability of gas-tight syringes as transfer/storage media for gaseous VOC analysis: The extent of VOC sorption between the inner needle and a glass wall surface. *Anal. Chem.* **87**, 3056-3063 (2015).
5. Kim, Y.-H., Kim, K.-H., Szulejko, J. E. & Parker, D. Development of the Detection Threshold Concept from a Close Look at Sorption Occurrence Inside a Glass Vial Based on the In-Vial Vaporization of Semivolatile Fatty Acids. *Anal. Chem.* **86**, 6640-6647 (2014).
6. Kim, Y.-H. & Kim, K.-H. Novel approach to test the relative recovery of liquid-phase standard in sorbent-tube analysis of gaseous volatile organic compounds. *Anal. Chem.* **84**, 4126-4139 (2012).
7. Kim, Y.-H. & Kim, K.-H. Ultimate detectability of volatile organic compounds: how much further can we reduce their ambient air sample volumes for analysis? *Anal. Chem.* **84**, 8284-8293 (2012).

Supplementary figure legends

Figure S1 | Illustration of the sampling methods for the three types of EC samples using the sorbent tube. [A] Liquid sample (S_E and S_S). [B] Vapor sample (V_E and V_S). [C] Aerosol sample (A_E and A_S).

Figure S2 | Chromatograms of the carboxyl compounds in the EC samples between TIC and SIM modes.

Table S1 | Calibration and QA results using the liquid working standards of the VOC: (1) RF, (2) R2, (3) RSE, and (4) MDL.

Order	Compound	[A] Response factor (RF, ng^{-1})	[B] Coefficient of determination (R^2)	[C] Relative standard error[a] (RSE, %)	[D] Method detection limit[b] (MDL, ng)
1	BA	**47,968**	0.9934	1.36	0.045
2	IA	**49,068**	0.9981	0.55	0.056
3	VA	**44,056**	0.9979	0.42	0.060
4	MEK	**60,840**	0.9974	0.79	0.043
5	MIBK	**58,667**	0.9982	0.72	0.038
6	BuAc	**60,487**	0.9967	0.85	0.043
7	i-BuAl	**50,593**	0.9973	0.81	0.044
8	B	**75,337**	0.9953	0.32	0.036
9	T	**114,862**	0.9920	1.71	0.014
10	p-X	**139,622**	0.9962	0.84	0.019
11	m-X	**126,940**	0.9952	0.70	0.026
12	o-X	**128,254**	0.9955	0.78	0.021
13	S	**127,074**	0.9943	1.40	0.014
14	PPA	**13,983**	0.9948	0.64	0.197
15	IBA	**13,103**	0.9948	1.05	0.108
16	BTA	**43,486**	0.9948	1.08	0.071
17	IVA	**37,553**	0.9948	1.25	0.077
18	VLA	**55,559**	0.9948	2.22	0.066
19	HXA	**53,277**	0.9948	1.95	0.068
20	HPA	**45,984**	0.9948	1.05	0.059
21	o-C	**78,123**	0.9948	2.80	0.041
22	m-C	**94,490**	0.9948	2.56	0.060
	Mean (n = 22)	-	0.9957	1.18	0.055
	SD (n = 22)	-	0.0017	0.68	0.039

[a]Triplicate analyses of the second calibration point (injection volume = 1 µL).

[b]The MDL values were determined using the heptaplicate analyses of the diluted final L-WS (mean 0.41 ± 0.11 ng μL^{-1}): (1) analytical volume = 1 µL and (2) concentration = mean 0.41 ± 0.11 ng μL^{-1}.

Table S2 | Basic information on the 22 target VOCs and the common solvents for the electronic cigarette (EC) solution in this study.

Order	Group	Compounds	Abbreviated Name	Molecular weight (g mol^{-1})	Density (g cm^{-3})	Boiling point (℃)	Formula	CAS number	Mass spectra[a] (m/z)
**[A] Target compounds**									
1	Aldehyde	n-Butyraldehyde	BA	72.11	0.8016	74.8	C_4H_8O	123-72-8	43, 44
2		i-Valeraldehyde	IA	86.13	0.785	92	$C_5H_{10}O$	590-86-3	41, 43, 44
3		n-Valeraldehyde	VA	86.13	0.8095	102-103	$C_5H_{10}O$	110-62-3	41, 43, 44
4	Ketone	Methyl ethyl ketone	MEK	72.11	0.8050	79.64	C_4H_8O	78-93-3	43, 72
5		Methyl isobutyl ketone	MIBK	100.16	0.802	117-118	$C_6H_{12}O$	108-10-1	43
6	Ester	n-Butyl acetate	BuAc	116.16	0.8825	126.1	$C_6H_{12}O_2$	123-86-4	43
7	Alcohol	i-Butyl alcohol	i-BuAl	74.122	0.802	107.89	$C_4H_{10}O$	78-83-1	41-43
8	Aromatic	Benzene	B	78.11	0.8765	80.1	C_6H_6	71-43-2	78
9		Toluene	T	92.14	0.87	111	C_7H_8	108-88-3	91, 92
10		p-Xylene	p-X	106.17	0.861	138.35	C_8H_{10}	106-42-3	91, 106
11		m-Xylene	m-X	106.17	0.86	139	C_8H_{10}	108-38-3	91, 106
12		o-Xylene	o-X	106.17	0.88	144.4	C_8H_{10}	95-47-6	91, 106
13		Styrene	S	104.2	0.909	145	C_8H_8	100-42-5	78, 103, 104
14	Carboxyl	Propionic acid	PPA	74.08	0.98797	141.15	$C_3H_6O_2$	79-09-4	73, 74
15		i-Butyric acid	IBA	88.11	0.9697 (0 ℃)	155	$C_4H_8O_2$	79-31-2	73
16		n-Butyric acid	BTA	88.11	0.9528	163.75	$C_4H_8O_2$	107-92-6	60, 73
17		i-Valeric acid	IVA	102.13	0.925	175-177	$C_5H_{10}O_2$	503-74-2	60
18		n-Valeric acid	VLA	102.13	0.930	186-187	$C_5H_{10}O_2$	109-52-4	60, 73
19		n-Hexanoic acid	HXA	116.16	0.929	205.8	$C_6H_{12}O_2$	142-62-1	60, 73
20		n-Heptanoic acid	HPA	130.18	0.9181	223	$C_7H_{14}O_2$	111-14-8	60, 73
21	Phenol	o-Cresol	o-C	108.14	1.0465	191	C_7H_8O	95-48-7	107, 108
22		m-Cresol	m-C	108.14	1.034	202.8	C_7H_8O	108-39-4	107, 108
**[B] Common solvents for e-cigarettes**									
1		Propylene glycol	PG	76.09	1.036	188.2	$C_3H_8O_2$	57-55-6	45
2		Glycerin (Vegetable)	VG	92.09	1.261	290	$C_3H_8O_3$	56-81-5	61

[a]Mass spectra selected for the EIC-base (or SIM-base) analysis

Table S3 | Information on the electronic cigarette (EC) device and EC liquid solution (S_E) used for the analysis in this study.

[A] Information on the EC device

Photo	Electronic cigarette (EC)	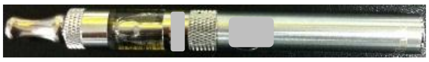
	Cartomizer	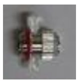
Company	Location	Seoul, Korea

[B] Information on the e-cigarette solution sample

1	Flavor:	Savory ciga	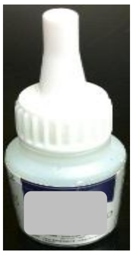
2	Solvent:	Propylene glycol and vegetable glycerin	
3	Nicotine content:	None	

Table S4 | Basic information regarding the preparation of liquid-phase VOC standards for TD-GC-MS analysis.

[A] Preparation of the two types of primary standards (PS-1 and PS-2): mixture of the reagent grade chemicals (RGC) in two separate (2 mL) vials

Order	Compounds	**RGC** (%)	**PS[a]-1** (ng µL^{-1})	Compounds	**RGC** (%)	**PS[a]-2** (ng µL^{-1})
a. Group 1: Aldehyde, Ketone, Ester, Alcohol, and Aromatic				*b. Group 2: Carboxyl and Phenol*		
1	BA	99.0	61,304	PPA	99.0	108,900
2	IA	97.0	59,468	IBA	99.0	106,667
3	VA	97.0	60,438	BTA	99.0	105,380
4	MEK	99.0	61,304	IVA	99.0	101,750
5	MIBK	99.5	61,384	VLA	99.0	103,180
6	BuAc	99.5	67,430	HXA	99.0	101,970
7	i-BuAl	99.0	60,999	HPA	99.0	100,991
8	B	99.5	67,201	o-C	99.0	115,115
9	T	99.5	66,282	m-C	99.0	113,740
10	p-X	99.0	65,873			
11	m-X	99.0	65,873			
12	o-X	97.0	65,662			
13	S	99.0	68,995			
Mixing recipe: in volume (µL)[b]:		RGC	each 150		RGC	each 200
		Total	1,950		Total	1,800

[B] Liquid working standard (L-WS): Mixture of PS-1, PS-2, and methanol

Order	Compounds		1st L-WS[c] (ng µL^{-1})		Final L-WS (ng µL^{-1}) 1st	2nd	3rd	4th
1	BA		61.3		3.07	14.6	30.7	61.3
2	IA		59.5		2.97	14.2	29.7	59.5
3	VA		60.4		3.02	14.4	30.2	60.4
4	MEK		61.3		3.07	14.6	30.7	61.3
5	MIBK		61.4		3.07	14.6	30.7	61.4
6	BuAc		67.4		3.37	16.1	33.7	67.4
7	i-BuAl		61.0		3.05	14.5	30.5	61.0
8	B		67.2		3.36	16.0	33.6	67.2
9	T		66.3		3.31	15.8	33.1	66.3
10	p-X		65.9		3.29	15.7	32.9	65.9
11	m-X		65.9		3.29	15.7	32.9	65.9
12	o-X		65.7		3.28	15.6	32.8	65.7
13	S		69.0		3.45	16.4	34.5	69.0
14	PPA		109		5.45	25.9	54.5	109
15	IBA		107		5.33	25.4	53.3	107
16	BTA		105		5.27	25.1	52.7	105
17	IVA		102		5.09	24.2	50.9	102
18	VLA		103		5.16	24.6	51.6	103
19	HXA		102		5.10	24.3	51.0	102
20	HPA		101		5.05	24.0	50.5	101
21	o-C		115		5.76	27.4	57.6	115
22	m-C		114		5.69	27.1	56.9	114
Mixing recipe in volume (µL)[b]:		PS-1 and-2	each 4	1st L-WS	100	500	1,000	2,000
		MeOH	3,992	MeOH	1,900	1,500	1,000	0
		Total	4,000	Total	2,000	2,000	2,000	2,000

[a] Primary standard (PS) used to prepare the liquid-phase working standard.

[b] Mixing conditions and formula of the PS and L-WS are given.

[c] The first liquid working standard (1st L-WS): Mixture of PS-1, PS-2, and methanol; Analysis (injection) volume for the sorbent tube loading was fixed at 1 µL.

Table S5 | Operational conditions for the analysis of the 22 target VOCs by the TD-GC-MS system.

**[A] Thermal desorber (Model: Unity II, Markes, UK)**	
a. Sampling tube	
1. Trap tube:	Quartz (length: 90 mm, OD: 6.4 mm, and ID: 4.2 mm)
2. Adsorbent:	3 bed: Carbopack C (70 mg), B (50 mg), and X (50 mg)
3. Desorption flow:	100 mL min^{-1} (to cold-trap)
4. Desorption time:	5 min
5. Desorption temp.:	300℃
b.Cold-trap	
1. Trap tube:	Quartz (length: 100 mm, OD: 3.2 mm, and ID: 2 mm)
2. Adsorbent:	Carbopack C and B (volume ratio = 1:1)
3. Adsorption temp.:	0℃ (from sampling tube)
4. Desorption temp.:	300℃ (to GC)
c.Carrier gas setting	
1. Carrier gas:	Helium (> 99.999%)
2. Initial gas flow:	1.75 mL min^{-1}
3. Constant gas pressure:	25.0 psi
4. Initial split flow:	10 mL min^{-1}
d. Line and interface temp.: 200 ℃	

**[B] Gas chromatography (Model: Shimadzu GC-2010, Shimadzu, Japan)**	
a. Column:	CP-wax (Agilent J&W, USA)<br>(length: 60 m, diameter: 0.25 mm, and film thickness: 0.25 μm)
b. Oven setting:	40 ℃ (10 min) → 10 ℃/min → 220℃ (10 min)<br>(Total program time = 38 min)

**[C] Mass spectrometry (Model: Shimadzu GCMS-QP2010 ultra, Shimadzu, Japan)**	
a. Ionization mode:	EI (70 eV)
b. Ion source temp.:	230℃
c. Interface temp.:	230℃
d. TIC mode:	35~500 m/z (0~24 mins)
e. SIM mode:	60, 73, 74, 107, and 108 m/z (24~ 38 mins)

Table S6 | Results of the analysis of the three different types of EC samples: (1) Liquid, (2) Vapor, and (3) Aerosol.

Order	Sample code:	[A] Concentration[a] S_E	V_E	A_E		S_S[b]	V_S[b]	A_S[b]		[B] Coefficient of determination (R^2) V_E	A_E		V_S	A_S	
	Mass per volume:	$(\frac{VOC\ (\mu g)}{S_E\ (mL)})$	$(\frac{VOC\ (\mu g)}{V_E\ (m^3)})$	$(\frac{VOC\ (\mu g)}{S_E\ (mL)})$	$(\frac{VOC\ (\mu g)}{A_E\ (m^3)})$	$(\frac{VOC\ (\mu g)}{S_S\ (mL)})$	$(\frac{VOC\ (\mu g)}{V_S\ (m^3)})$	$(\frac{VOC\ (\mu g)}{S_S\ (mL)})$	$(\frac{VOC\ (\mu g)}{A_S\ (m^3)})$	$(\frac{VOC\ (\mu g)}{V_E\ (m^3)})$	$(\frac{VOC\ (\mu g)}{S_E\ (mL)})$	$(\frac{VOC\ (\mu g)}{A_E\ (m^3)})$	$(\frac{VOC\ (\mu g)}{V_S\ (m^3)})$	$(\frac{VOC\ (\mu g)}{S_S\ (mL)})$	$(\frac{VOC\ (\mu g)}{A_S\ (m^3)})$
1	BA	0.045	1.29	0.127	66.3	1.94	58.1	8.85	4,630	0.9544	0.9843	0.9792	0.9786	0.9928	0.9892
2	IA	0.056	0.56	0.022	11.2	2.27	90.0	16.5	8,640	-	-	-	0.9915	0.9975	0.9952
3	VA	0.060	0.60	0.024	12.0	3.52	76.0	15.0	7,830	-	-	-	0.9905	0.9882	0.9837
4	MEK	0.172	8.52	0.398	208	2.43	213	5.97	3,130	0.8577	0.9568	0.9487	0.9932	0.9974	0.9952
5	MIBK	0.038	0.38	0.015	7.60	2.16	250	12.8	6,700	-	-	-	0.9911	0.9977	0.9992
6	BuAc	0.043	3.98	0.098	51.5	2.43	368	13.2	6,940	0.9460	0.9920	0.9950	0.9954	0.9997	0.99999
7	i-BuAl	0.044	0.44	0.017	8.86	3.88	162	1.37	723	-	-	-	0.9654	0.8858	0.8976
8	B	0.036	8.59	0.167	87.5	2.11	283	15.7	8,230	0.9990	0.9997	0.9987	0.9904	0.9855	0.9897
9	T	0.059	20.2	0.337	176	1.97	436	21.2	1.11E+4	0.9791	0.9351	0.9254	0.9927	0.9865	0.9905
10	p-X	0.019	2.60	0.030	15.7	2.03	419	15.0	7,850	0.9956	0.9865	0.9817	0.9961	0.9978	0.9992
11	m-X	0.026	4.12	0.051	26.5	2.46	437	16.7	8,770	0.9948	0.9123	0.9013	0.9945	0.9983	0.9995
12	o-X	0.021	3.25	0.036	18.8	2.43	395	14.4	7,540	0.9971	0.9955	0.9926	0.9954	0.9994	0.99997
13	S	0.014	2.93	0.040	20.8	2.41	346	10.8	5,690	0.9994	0.9902	0.9936	0.9924	0.9979	0.9993
14	PPA	0.359	1.97	2.289	1,200	10.7	14.7	10.7	5,590	-	0.9997	0.99999	0.9712	0.9938	0.9904
15	IBA	0.379	1.08	5.849	3,070	10.4	14.9	10.4	5,430	-	0.9995	0.9982	0.9779	0.9668	0.9596
16	BTA	NA	0.71	NA	NA	NA	0.71	NA	NA	-	-	-	-	-	-
17	IVA	0.077	0.77	0.030	15.4	9.14	0.77	7.72	4,030	-	-	-	-	0.9618	0.9541
18	VLA	0.066	0.66	0.309	162	10.1	0.66	10.1	5,290	-	0.9917	0.9879	-	0.9959	0.9932
19	HXA	0.068	0.68	1.506	794	10.2	0.68	9.89	5,170	-	0.9069	0.9176	-	0.9676	0.9605
20	HPA	0.059	0.59	0.023	11.9	10.5	0.59	9.90	5,180	-	-	-	-	0.9938	0.9904
21	o-C	0.041	0.41	0.016	8.12	11.6	0.41	11.3	5,940	-	-	-	-	0.9951	0.9921
22	m-C	0.060	0.60	0.024	12.0	11.3	0.60	11.1	5,830	-	-	-	-	0.9959	0.9931
23	ACA	25.8	169	166	8.72E+4	-	-	-	-	0.9985	0.9951	0.9920	-	-	-

NA Not available because of the solvent effect

[a]The concentrations were calculated based on the linear regression analysis between detected mass (y-axis) and sample volume (or mass) (x-axis).

[b]The quantity of target compounds in spiking standards is adjusted by subtracting those contained in the environmental EC samples (S_E, V_E, and A_E).

Figure S1 | Illustration of the sampling methods for the three types of EC samples using the sorbent tube.

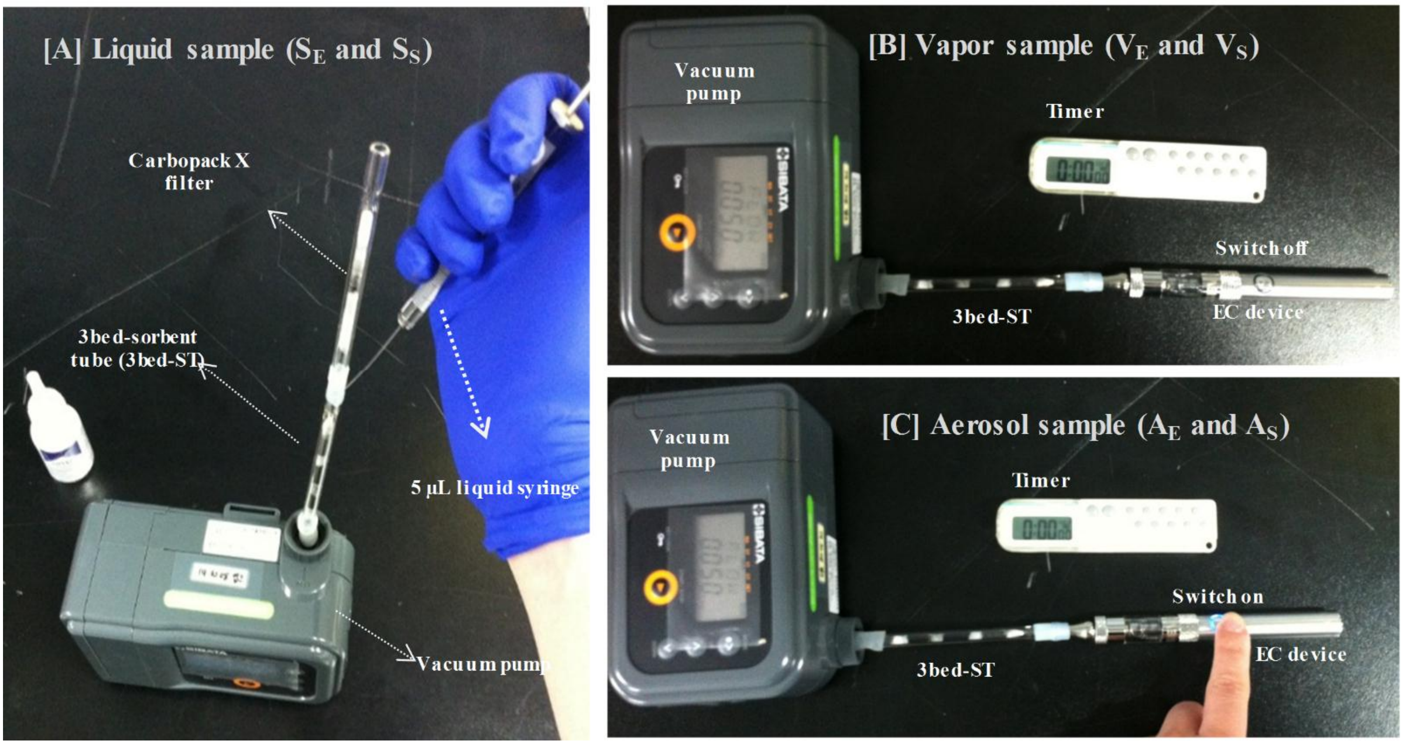

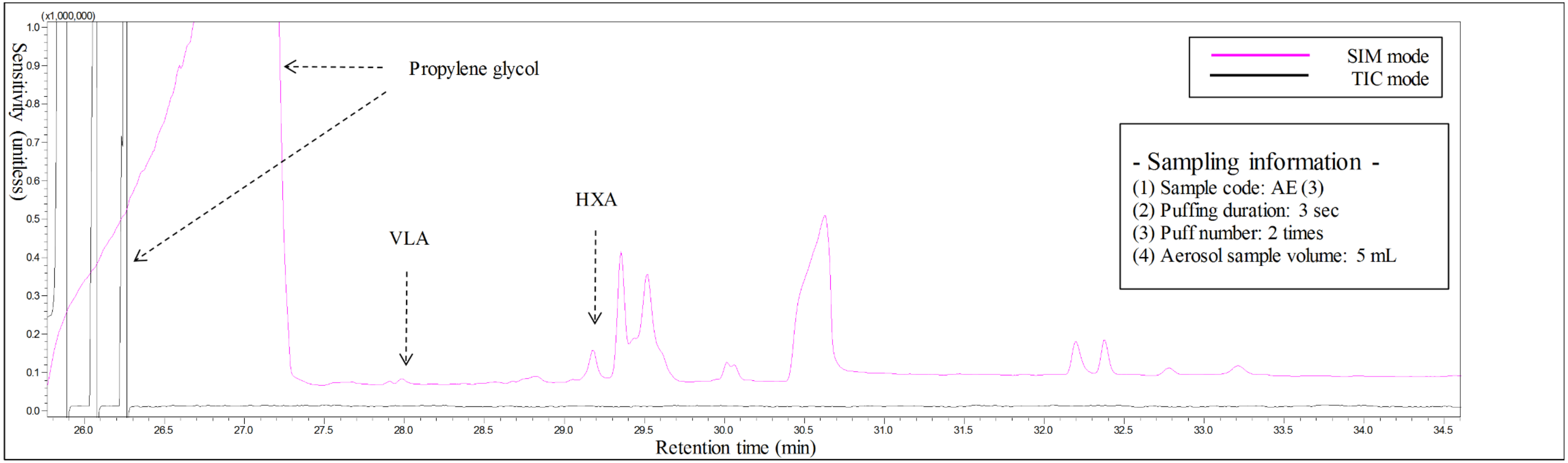

Figure S2 | Chromatograms of the carboxyl compounds in the EC samples between TIC and SIM modes.
